# In silico Study of *Trianthema portulacastrum* Embedded Iron Oxide Nanoparticles on Glycogen Synthase Kinase-3β: A Possible Contributor to its Enhanced *in vivo* Wound Healing Potential

**DOI:** 10.3389/fphar.2021.664075

**Published:** 2021-05-17

**Authors:** Ekta Yadav, Pankajkumar Yadav, Amita Verma

**Affiliations:** ^1^ Bioorganic and Medicinal Chemistry Research Laboratory, Department of Pharmaceutical Sciences, Sam Higginbottom University of Agriculture, Technology and Sciences (SHUATS), Prayagraj, India; ^2^ Pharmaceutics Laboratory, Department of Pharmaceutical Sciences, Sam Higginbottom University of Agriculture, Technology and Sciences (SHUATS), Prayagraj, India

**Keywords:** ointment, antioxidant, wound healing, nanoparticles, iron oxide

## Abstract

Rich amount of phenolic compounds are available in *Trianthema portulacastrum* L. (TP) leaves and are traditionally utilized as a wound dressing material. Oxidative stress and inflammation affect the Wnt/β-catenin pathway by modulating the glycogen synthase kinase-3β (GSK) activity subjected to delay in wound healing. The objective of the current study was to explore the wound healing effect of ferric oxide nanoparticles biosynthesized with fractionated TP extract (FeTP). The ability of TP active components (polyphenols) to inhibit the GSK was explored by using molecular docking studies. FeTP were synthesized, characterized, utilized to prepare an ointment and its efficacy was investigated against full-thickness dermal wounds. Different wound healing parameters, level of enzymatic antioxidants, hydroxyproline content and tissue cytokines level were analyzed. Histopathology was performed to confirm the healing by newly formed tissue architecture. Rats treated with FeTP showed significantly swift healing with faster wound contraction rate, high tensile strength and hydroxyproline content along with the utilization of less time for epithelialization. Histopathological study also validated the potential wound healing effect of FeTP with complete re-epithelialization. The results of the present study cumulatively revealed that the green synthesized FeTP ointment approach may serve as a potential tool for dermal wound healing.

## Introduction

Skin is the largest protective barrier of the body, which provides primary protection from the environment. Any physical, chemical and thermal damages on the subcutaneous tissue cause wound formation due to loss of dermal integrity (Bowler et al., 2001). Healing of wound is a complicated pathophysiological mechanism involving various overlapping phases such as inflammatory, proliferative and maturation as well as remodeling phase to accomplish the process of tissue repairs. Along with this, different biochemical and cellular pathways and various enzymatic pathways also take place to restore the structure of damaged cutaneous as well as subcutaneous tissues (Gonzalez et al., 2016). Upon onset of inflammatory phase, the incursion of inflammatory cells and local Wnt/β-catenin signaling pathway starts to accelerate. Basically, Wnt proteins are glycoproteins that aid to control the process of proliferation, differentiation, migration and determination of cell fate (Harish et al., 2008). Wnt/β-catenin pathway further promotes wound repairing by inhibiting an essential responsible regulatory enzyme, i. e. glycogen synthase kinase-3β (GSK) (Vidya et al., 2012). It promotes the proliferative phase characterized by initiation of re-epithelialization of wound area with the formation of eschar. In this phase, deposition of cytoplasmic β-catenin takes place which causes the matrix metalloproteinase gene transcriptions leading to angiogenesis, accumulation of extracellular matrix, different cellular components formation and proliferation including fibroblasts and keratinocytes (Cheon et al., 2004; Kulac et al., 2013). The maturation and remodeling phase direct complete re-epithelialization of epidermis to recuperate the function of protective barrier. During this phase, transforming growth factor-β (TGF-β) protein is attributed to complete wound healing via proliferation of fibroblast, angiogenesis, collagen organization and remodeling of extracellular matrix (Wang et al., 2006). Previous studies have revealed that various biological hallmarks are responsible for the process of faster wound healing including reduction of oxidative stress, inflammatory cytokines synthesis as well as inflammatory transduction cascades. Whereas an increase in the level of enzymatic antioxidants, acceleration of neovascularization and angiogenic pathways help in swift wound healing. (Zhang et al., 2009a; Vidya et al., 2012). Since delay in wound healing is attributed to an increased level of oxidative stress and pro-inflammatory cytokines which further cause reduction in growth factors and cellular signaling activities, therefore, strong antioxidant and anti-inflammatory activity bearing agent could serve the need (Mohanty et al., 2012; Jere et al., 2019).

Over recent years, nanomaterials-based therapy for wound healing has prevailed as a successful approach since it protects from bacterial infections as well as cell specificity, which was not possible with conventional means of wound healing (Naskar and Kim, 2020). Nanomaterials have already been drawing attention and utilized in various fields of biomedical such as biosensing, bioimaging, diagnosis, medical equipment, drug delivery as well as cosmetics (Jiao et al., 2018; Hu et al., 2019; Naskar and Kim, 2019). The widespread utilization of nanomaterials is mostly due to their unique physicochemical properties starts with the nanoscale size range, i. e. 1–100 nm, high surface energy, and large surface area to volume ratio along with optical and magnetic properties (Akbarzadeh et al., 2012). Especially regarding wound healing activity, relatively smaller size and high surface area to volume ratio render nanomaterials to penetrate and act more effectively with the region of injury. Therefore, these nanomaterials along with the healing effect work as a sustained release of therapeutic means at the site of the wound (Naskar and Kim, 2020). Among different metal oxide nanoparticles, iron oxide nanoparticles are well-known in the research field as they owe superparamagnetic properties that are allowed to be directed with an external magnetic field. Previous studies have reported that topically applied iron chelators and their novel therapeutic agents are beneficial in delayed wound healing (Wright et al., 2014). In the current scenario, due to the distinct surface chemistry of ferric oxide nanoparticles (FeNP), it has been intensively utilized in the different biomedical application such as cell separation, repairing of tissue, targeted drug delivery by magnetic system, bio-labeling material and MRI contrast enhancement (Nagajyothi et al., 2017; Devi et al., 2019). Whereas biofabricated FeNPs are also reported to exhibit good therapeutic effects, i. e. anticancer activity (Nagajyothi et al. (2017)), antifungal (Devi et al. (2019)), antimicrobial activity (Sharma et al. (2018)) and wound healing activity (Zangeneh et al., 2020)). Different methods (chemical, physical and biological) are used to synthesize various types of nanoparticles. But physical and chemical methods have inevitable limitations such as needs a high range of temperature, pressure and chemicals which toxically affect the environment as well as the biological system (Zangeneh et al., 2020). Therefore, biological method is a preferred cost-effective and eco-friendly method for the production of nanoparticles by using plant extracts, bacteria, fungus, etc., with the additional advantage of utilizing the existing phytoconstituents of plant extract as reducing and capping agent (Yadav et al., 2018c).

For the current study, we have selected *Trianthema portulacastrum* L (TP), a plant of Aizoaceae family, indigenous to Asia, tropical America and Southeast Africa. It grows like a weed, especially in areas with heavy rainfall and a good amount of irrigation in India, Sri Lanka, Pakistan and Bangladesh (Yadav et al., 2018a; Yadav et al., 2019). TP is also a well-known Indian traditional medicinal herb of the Ayurvedic system and it has been traditionally utilized for the treatment of broad-spectrum ailments such as jaundice, inflammation ulcer, anemia, liver disease and migraine. Different parts of TP possess a variety of chemical compounds such as phenolic compounds, flavonoids, terpenoids, glycosides, steroids and alkaloids (Shivhare et al., 2012). TP has been scientifically explored to have various therapeutic activities such as antifungal, anti-inflammatory, antioxidant, hepatoprotective, wound healing, hypolipidemic, antitumor, anthelmintic, etc (Yadav et al., 2017).

Molecular docking analysis is especially employed to determine the binding effect of desired small bioactive molecules with the backbone of the respective protein. In our previous study, we have reported that butanol fraction of TP hydroethanolic extract was found to be rich in phenolic compounds such as protocatechuic acid, chlorogenic acid, caffeic acid and ferulic acid, which was individually quantified by high-performance liquid chromatography with diode array detector (HPLC-DAD) analysis (Yadav et al., 2017). The current study explores the binding potential of those identified phenolic compounds with the targeted protein, i. e. GSK, in silico. This research work aimed toward green synthesis, characterization and evaluation of wound healing potential of FeNPs of TP leaves extract butanol fraction (FeTP).

## Material and Methods

### Chemicals and Reagents

Acetic acid, sodium hydroxide, sodium chloride, hydrogen peroxide, sodium azide, hydrochloric acid, and perchloric acid were purchased from Qualigens (Mumbai, India). Ascorbic acid, nitro blue tetrazolium (NBT) and ethylene diamine tetra acetic acid (EDTA) were procured from Hi Media Laboratories Pvt. Ltd. Mumbai, India. 2, 2-diphenyl-1-picrylhydrazyl (DPPH) and 5, 5^′^-dithio-bis-2-nitrobenzoic acid (DTNB) were purchased from Sigma-Aldrich (St. Louis, Missouri, United States). All chemicals utilized in the present study were of HPLC and analytical grade.

### Plant Collection and Authentication

TP leaves were collected in the rainy season from the rural region of Rewari (Haryana), identified and authenticated by botanist Dr. R. M. Kadam (Department of Botany, Mahatma Gandhi Mahavidyalaya, Latur, Maharashtra, India). The voucher specimen has been submitted to the herbarium collection.

### Preparation and Liquid–Liquid Fractionation of Hydroethanolic Extract

To prepare the hydroethanolic extract, fresh TP leaves (2.5 kg) were shade dried and reduced to a coarse powder in an electric blender. Leaf powder was defatted with petroleum ether (60–80°C) and then macerated with the water-ethanol mixture (ethanol: water, 70:30) for three times. The macerated mixture was subjected to filtration by using Whatman filter paper. Residue was dried under vacuum and stored as hydroethanolic extract of TP (HETP, 112 g).

Liquid-liquid partitioning of HETP was performed to increase the solvent polarity to get different fractions. The aqueous solution of HETP was partitioned with chloroform, ethyl acetate and n-butanol, respectively, using a separating funnel. The resultant butanol fraction (BuTP, 45.4 g) was collected and preserved at −20°C till further study. Quantitative analysis of phenolic compounds (i. e. protocatechuic acid, cholorogenic acid, ferulic acid and caffeic acid) in BuTP was performed by using HPLC (Shimadzu, Japan) equipped with a diode array detector (DAD-MZOA) (Yadav et al., 2017).

### Molecular Docking Studies

Crystal structure of GSK (Protein Data Bank ID: 1Q5K), involved in Wnt/β-catenin pathway, was downloaded from Research Collaboratory for Structural Bioinformatics (RCSB) website. Autodock 4.2 (The Scripps Research Institute) was used for molecular docking studies of protocatechuic acid, cholorogenic acid, ferulic acid and caffeic acid with GSK. Protein was prepared by removal of water molecules, repairing missing atoms, addition of polar hydrogens and Kollman charge. The active site in 1Q5K crystal was demarcated in accordance with the binding site of co-crystallized inhibitor with drug bank ID DB01950 and the receptor grid was generated by using XYZ coordinates of 60 × 60 × 60 with a grid spacing of 0.375 Å. Docking was performed by using following parameters: number of runs: 50, population size: 300, number of evaluations: 2,500,000 and number of generations: 27,000. Discovery Studio Visualizer was used for visualization and further analysis of ligand-protein interactions.

### Green Synthesis of FeTP

FeTP were biofabricated according to the previously reported method with slight modification (Makarov et al., 2014). BuTP was slowly added into 0.1 M ferric chloride hexahydrate solution (FeCl_3_.6H_2_O) in 2: 1 ratio v/v, respectively and the reaction was completed with mixing of 0.1 M sodium hydroxide solution. Mixture was stirred continuously on a magnetic stirrer at 70°C for 2 h. Synthesis of nanoparticles was confirmed by the appearance of intense brown color from yellow due to the reduction of ferric ions into FeNPs. The mixture was centrifuged at 12,000 rpm for 20 min. After washing and drying, nanoparticles were separated and preserved in a vacuum desiccator as FeTP till further study.

### Characterization of Biosynthesized Nanoparticles

#### UV Spectrophotometry

UV-Visible spectrophotometer (Shimadzu UV-1800, Japan) was used to detect the optical characteristics of green synthesized FeTP. The sample was suspended in sterile de-ionized water for scanning.

#### Fourier Transform Infrared

FT-IR spectrophotometer (Perkin-Elmer Spectrum 1,000) was employed to confirm the existence of active phytoconstituents of BFTP responsible for reduction and stabilization of prepared metal oxide nanoparticles. FT-IR spectra of BFTP and FeTP were recorded at a wavelength range of 4,500–400 cm^−1^.

#### Field Emission Scanning Electron Microscopy

Surface morphology along with metal analysis of FETP was observed with FESEM (Carl Zeiss, Germany) fabricated with energy-dispersive X-ray (EDX) spectroscopy (EDX-JEOL, JSM-5610). FESEM analysis was performed by spraying the dry sample (nanopowder) over the carbon tape followed by gold coating.

### DPPH Radical Scavenging Activity

To assess the hydrogen or electron donation ability of FeTP for the antioxidant effect, DPPH radical scavenging activity was performed according to a previously published slightly modified assay (Kedare and Singh, 2011). Antioxidant reaction was initiated with 150 µL of 3.3 mM DPPH solution mixed into 100 µL of different concentration FeTP solution as well as ascorbic acid (standard) solution, prepared from their respective stock solution. The resultant solutions were allowed to incubate for 30 min in darkness at 25°C and then each sample was spectrophotometrically scanned at 517 nm. Radical scavenging activity was measured by following formula:

$$\mathrm{Free radical scavenging activity(\%)}\;=\frac{\;\mathrm{Absorbance of control-Absorbance of sample}}{\mathrm{Absorbance of control}}\mathrm{\times 100}$$

### Preparation of FeTP Ointment

For the preparation of a simple ointment base, wool fat: hard paraffin: cetostearyl alcohol: white soft paraffin (1:1:1:17) were used. Firstly, hard paraffin and cetostearyl alcohol were allowed to melt in a china dish with continuous stirring on water bath (60°C), then white soft paraffin was mixed followed by wool fat at a slow rate to get a homogenous mixture with a smooth consistency. Upon cooling, green synthesized FeTP (20 mg/g) and Fe_3_O_4_ (20 mg/g) were uniformly mixed with simple ointment base separately by using a homogenizer.

### Animals

Healthy male albino Wistar rats (180 ± 20 g) were selected and acclimatized to standard laboratory conditions of temperature along with humidity for one week before performing wound healing activity. They were allowed to get water ad libitum and provided with a standard food pellet diet during the whole experimentation period. Experimental protocol was approved by the Institutional Animal Ethical Committee (IAEC).

### Acute Dermal Toxicity Test

To assess the skin irritation and other toxicities of the test sample, acute dermal toxicity assay was conducted prior to the main activity evaluation as per the Organization for Economic Cooperation and Development (OECD) guidelines 402 at a dose level of 200 mg/kg. Test ointments were regularly applied topically to the pre-shaved dorsal area of rats for the next 15 days and daily checked for adverse skin reactions such as itching, erythema, inflammation and irritation as compared to control group.

### Animal Grouping for *in vivo* Wound Healing Activity

Incision and excision wound models were utilized to explore the dermal wound healing potential of sample ointment (FeTP). Animals were randomly divided into four groups and each group comprised of six animals.

Group 1 (control group): applied with simple ointment base, group 2 (standard group): applied with standard drug ointment, i. e., povidone-iodine ointment USP (10% w/w), group 3 (FeBL group): applied with Fe_3_O_4_ (2% w/w) ointment, group 4 (FeTP group): applied with FeTP (2% w/w) ointment. All rats were treated topically with their respective drug treatment once a day till complete healing of the injured area was observed.

### Excision Wound Model

A wound of 500 mm^2^ area and 2 mm thickness was made at the pre-shaved dorsal area of rats by excising the skin with the help of a sharp sterile surgical scissor after providing mild anesthesia (Yadav et al., 2018b). The created wound area was immediately cleaned with a cotton swab dipped in normal saline and then respective treatment was provided from day 0 till complete healing. Different parameters (rate of wound contraction, epithelialization period and hydroxyproline content were observed to estimate the wound healing potential of test sample.

#### Collection of Blood and Tissue

On the last day of experimental work, granulation tissues were collected from euthanized rats, preserved and further subjected to perform various biochemical assays as well as histological examination. Levels of antioxidants and proinflammatory cytokines were estimated in granulation tissue which was homogenized (50 mM Tris-HCl: 150 mM NaCl in a ratio of 1:2, pH 7.4, Ultra-Turax homogenizer), then the supernatant was collected after centrifugation (15 min, 5,500 rpm, 4°C) and stored at −80°C till further study.

### Determination of Wound Healing Parameters

#### Wound Contraction Rate

On every third day, wound area was marked on transparent paper and measured on a 1 mm^2^ graph sheet Wound contraction rate was calculated by using the below-given formula (Manjunatha et al., 2005):

$$\mathrm{Wound contraction rate (\%)=}\frac{\mathrm{Area of wound on day 0-Area of wound on day n}}{\mathrm{Area of wound on day 0}}\mathrm{\times 100}$$

Where, *n* denotes the number of days (3rd, 6th, 9th, 12th and 15th).

#### Hydroxyproline Estimation

After washing (cold saline) and drying (60°C) the granulation tissue, it was employed for acid hydrolysis with 6 N HCl for 4 h at 130°C. The hydrolyzed tissue was neutralized with 10 N sodium hydroxide and left for about 20 min to accomplish the chloramine-T oxidation reaction. Thereafter, perchloric acid (0.4 M) and Ehrlich reagent were added into the reaction mixture and spectrophotometrically scanned at 557 nm (Shimadzu UV-1800, Japan). The calibration curve of standard hydroxyproline was used to draw the results in mg/g of dry granulation tissue (Dwivedi et al., 2017).

#### Antioxidant Profile Estimation

Granulation tissue supernatant was utilized to estimate the level of antioxidant enzymes superoxide dismutase (SOD), catalase (CAT) and glutathione peroxidase (GPx). SOD activity was determined by following the previously described method based on the principle of diminishing photoreduction of NBT dye with the effect of SOD enzyme determined by analyzing the sample mixture at 560 nm (Winterbourn et al., 1975).

CAT activity was measured by using method reported by Sinha (Sinha, 1972). Absorbance of solution was measured at 570 nm and results were represented as μM of H_2_O_2_ consumed/mg protein in granulation tissue.

GPx enzyme level was determined by measuring the color intensity of solution obtained, after following the method described by Flohé and Günzler, at 412 nm and expressed as µM/mg protein in granulation tissue (Flohé and Günzler, 1984).

#### Tissue Cytokines Determination

The supernatant of collected granulation tissue was utilized for analysis of interleukin-6 (IL-6), tumor necrosis factor-α (TNF-α) and interleukin-10 (IL-10) status in different animal groups. Proinflammatory cytokines (IL-6 and TNF-α), as well as an anti-inflammatory cytokine (IL-10), were estimated with enzyme-linked immunosorbent assay by using a commercially available kit (R&D Systems, United States).

### Histology of Wound Tissue

Skin sample from the healed area was collected and stored 10% formalin solution for 24 h. Thereafter, the sample was dehydrated, cleaned and entrenched in paraffin wax. A section of 5 μm thickness was cut and dyed with hematoxylin and eosin and observed under a microscope.

### Incision Wound Model

A longitudinal line of 6 cm was marked and incised (2 mm) parallel to the paravertebral region on either side of the vertebral column on pre shaved dorsal area of rats under the influence of anesthesia. After cleaning the incised area, the incision was immediately sutured at a distance of 1 cm and carefully tied to both ends. After 24 h, topical treatment was provided with their respective drug regimen on the sutured site once a day, till complete healing was achieved. On 10^th^ day post-wounding, sutures were carefully removed but the drug was continuously applied and tensile strength of the healed area was observed with constant water flow method on 13th day (Ilango and Chitra, 2010).

### Statistical Evaluation

Data were expressed as mean ± standard error of mean (SEM). One-way analysis of variance (ANOVA) followed by Dunnett’s test was performed to compare the results. *p* < 0.05 was considered as statistically significant.

## Results and Discussion

### Molecular Docking Studies

**FIGURE 1:**
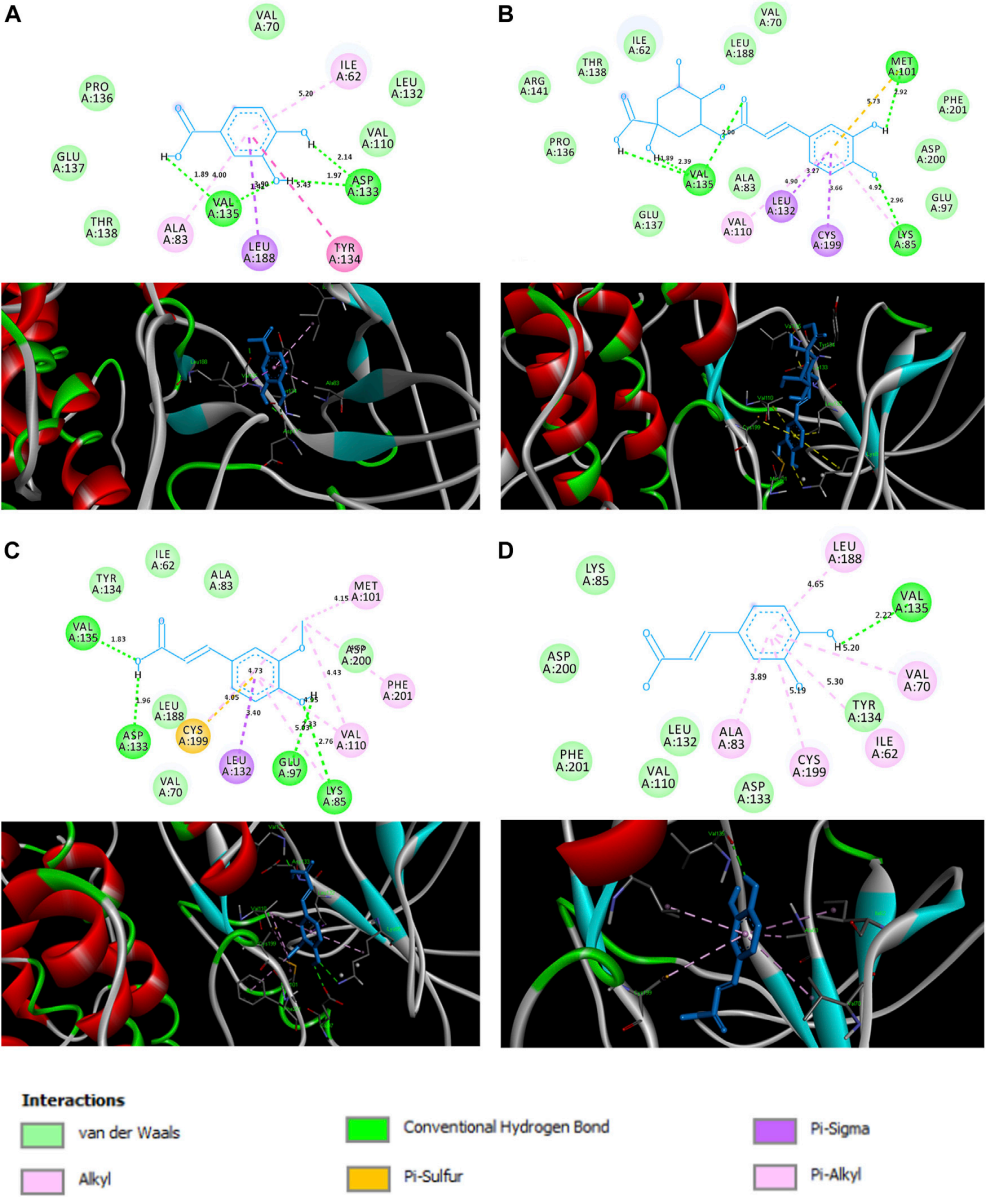
Interactions between **(A)** protocatechuic acid, **(B)** chlorogenic acid, **(C)** ferulic acid and **(D)** caffeic acid, and the amino acids of the active site of GSK (PDB ID: 1Q5K) illustrated in 2D **(top)** and 3D **(bottom)**.

Docking score analysis revealed that chlorogenic acid exhibited maximum negative binding score among all the ligands, which indicates its highest binding affinity toward GSK followed by ferulic acid, caffeic acid and protocatechuic acid (Table 1). 3D and 2D analysis of ligand and GSK interactions (e. g. hydrogen bonds, hydrophobic interactions, van der Waals, etc.) are shown in Figure 1. Protocatechuic acid showed hydrogen bond formation with Val 135 and Asp 133 residues of GSK. Chlorogenic acid displayed hydrogen bond formation with Val 135, Met 101 and Lys 85. Ferulic acid formed hydrogen bonds with Val 135, Asp 133, Glu 97 and Lys 85. Caffeic acid exhibited hydrogen bond formation with Val 135.

**TABLE 1:** Docking score of GSK active site with phenolic compounds.

**Compound**	**Docking score (kcal/mol)**
Protocatechuic acid	−6.59
Chlorogenic acid	−11.05
Ferulic acid	−7.71
Caffeic acid	−7.59

### Characterization of Biofabricated Nanoparticles

#### UV Spectrophotometry

**FIGURE 2:**
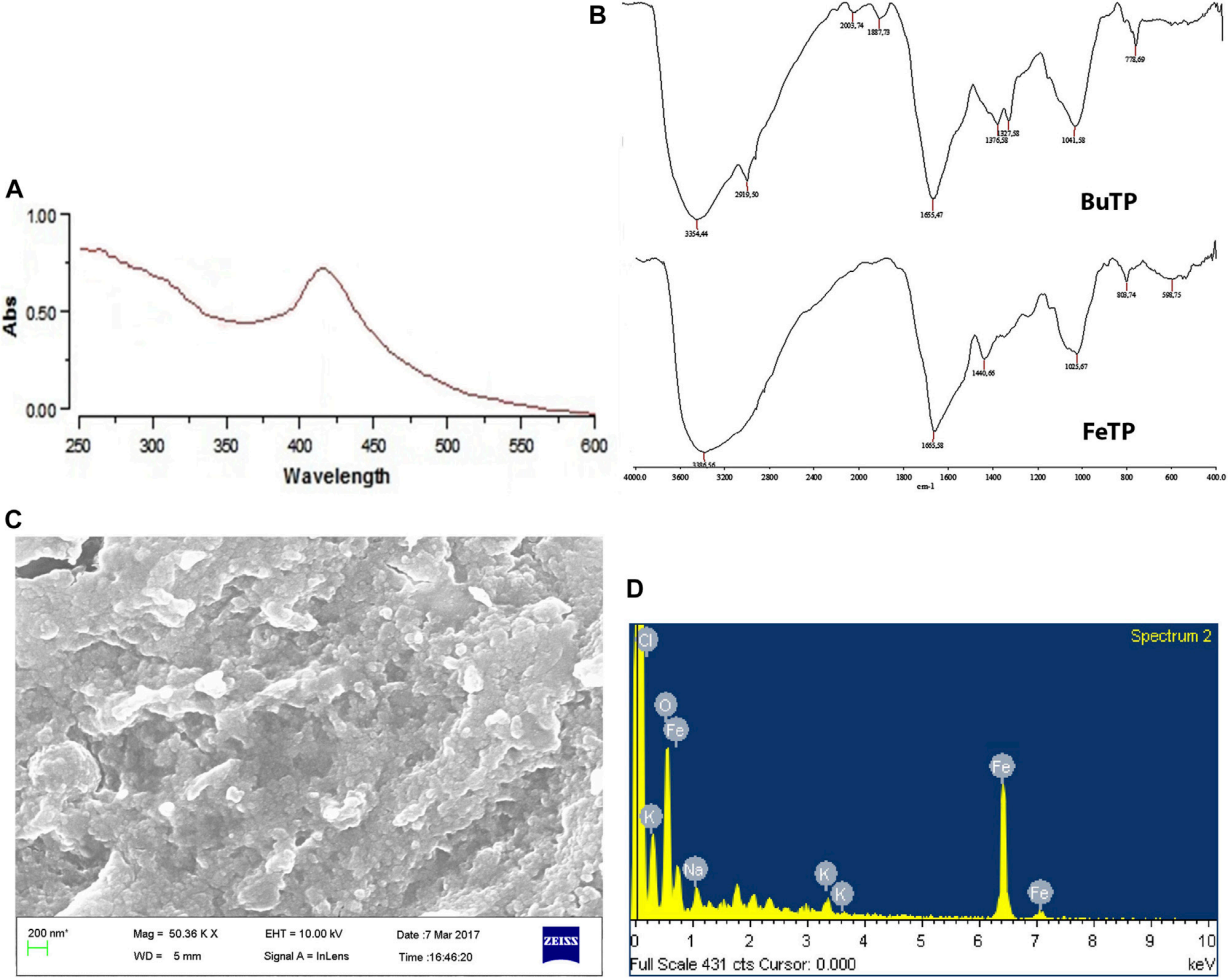
Characterization of green synthesized FeTP by **(A)** UV Visible spectra, **(B)** FT-IR (Top: BuTP, Bottom: FeTP), **(C)** FESEM micrograph, and **(D)** EDX spectrum.

Reduction of iron ions, as well as the formation of stable FeNPs, were primarily seen with the appearance of dark brown color from light yellow in the reaction mixture. It was further confirmed by UV–visible absorption spectra as it exhibited a sharp characteristic absorption peak at 412 nm (Figure 2A). Mie’s theory demonstrated that spherical or quasi-spherical nanocrystals show a single surface plasmon resonance band while two or three bands are related to shape formed by anisotropic particles (Fan et al., 2014). The absorption occurs due to the electronic transition of molecules in the electromagnetic region of the spectrum. It indicates well coordination and reduction of Fe^3+^ ion by various functional groups (hydroxyl, aldehyde, etc.) existed in phytoconstituents as well as stabilization of nanoparticles (Mahdavi et al., 2013). The result is concordant with past reports that suggest the absorption of super magnetic iron oxide nanoparticles coated with folate conjugated polymer occurs within the visible region (Zhang et al., 2009).

#### FT-IR

FT-IR spectra of BuTP exhibited the presence of various functional groups pertinent to its phytoconstituents (Figure 2B) with characteristic absorption band at 3,354.44 and 2,919.50 cm^−1^assigned to–OH stretching and–CH stretching while a peak at 1,887.73 cm^−1^ represents the non-H-bonded species water molecules. Other peaks were observed in FT-IR spectra of BuTP at 1,655.47 (C=C group), 1,376.58 (C-N stretching or O-H bending), 1,327.58 [C-O(H) stretching], 1,041.58 (C=O stretching) and 778.6 cm^−1^ (cis = C-H out of plane bending). After the synthesis of FeTP, the possible interaction of functional groups of TP in the biosynthesis of FeNPs was confirmed through the presence of various strong absorption bands (3,386.56, 1,665.58, 1,440.66, 1,025.67, 803.74 and 598.75 cm^−1^) in the FT-IR spectra (Figure 2B). The characteristic absorption bands at 3,386.56, 1,665.58 and 1,440.66 cm^−1^ were accounted to -OH stretching vibration in response to hydroxyl group of existed polyphenols and aromatic heterocyclic organic compounds of TP, conjugated carbonyl (-C=O) group stretching vibration and symmetric stretching of COO^−^, respectively. The formation of FeNP was further indicated by the presence of a characteristic absorption band at 598.75 cm^−1^ which is primarily associated with the intrinsic stretching vibration mode of metal oxide (Fe–O) bond in magnetite (Khatami et al., 2017). A shift in the position of absorption bands demonstrated the considerable contribution of various bioactive secondary metabolites of TP in FeNP stability (Yew et al., 2016). Since BuTP is rich in protocatechuic acid, chlorogenic acid, caffeic acid and ferulic acid, they could play a vital role in reducing and stabilization of FeNPs as a capping agent during biosynthesis.

#### FESEM

The FESEM image showed that FeTPs were almost spherical with the size range of 80–100 nm and present in cluster form as shown in (Figure 2C). In the EDX spectra (Figure 2D), different optical absorption peaks around 0.8, 6.2 and 7.1 keV are concerned with binding energies of Fe, whereas oxygen binding energy was found to be at 0.6 keV due to reflection of X-rays in response to interaction with metal compounds. EDX spectrum is useful in confirmation of purity and stability of synthesized FeNPs. Several other weak peaks were also observed due to the adsorption of other phytoconstituents on FeTP surface (Solomon et al., 2006). The current result is in good agreement with the previous study which suggested the formation of surface-modified spherical and stable biofabricated FeNPs by direct reduction (Latorre-Sanchez et al., 2012).

### Antioxidant Activity

**FIGURE 3:**
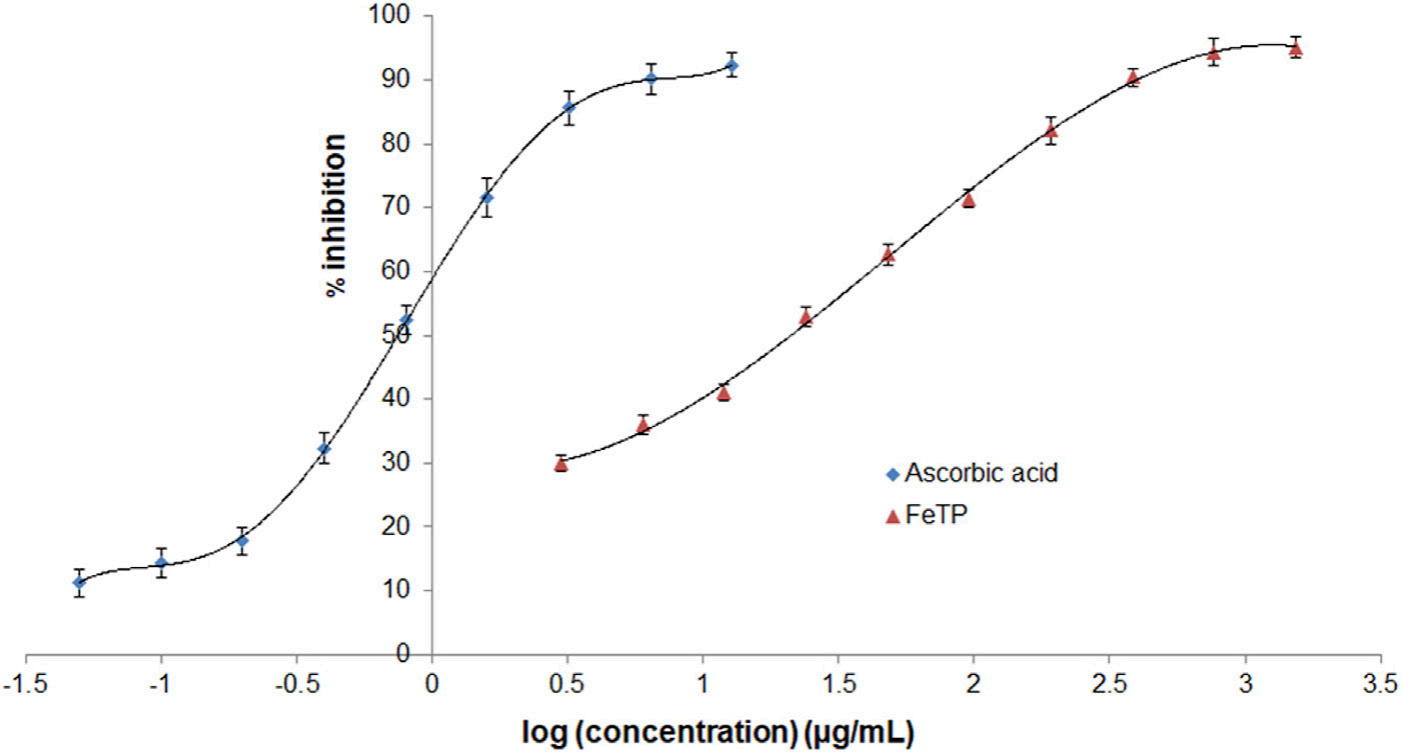
Comparative antioxidant activity of FeTP and ascorbic acid by DPPH assay. Data expressed as mean ± SD (n = 3).

The ability of FeTP to scavenge free radicals was estimated with DPPH method by assessing its bleaching effect toward stable radicals of DPPH. This method is based on the principle of reduction in absorption intensity and formation of pale yellow color from violet via reducing the DPPH to hydrazine upon addition of hydrogen donor substance (Formagio et al., 2014). During the onset of oxidative stress conditions, there is an imbalance between the cellular status of reactive oxygen species (ROS) and antioxidant system (Zangeneh et al., 2020). These free radicals are responsible for various chronic illnesses attributed to inflammation such as delay in wound healing as well as neurodegenerative diseases (Okur et al., 2018). FeTP showed a remarkable antioxidant effect with IC_50_ 21.12 μg/ml (Figure 3). It confirmed the strong electron or hydrogen atom donating effect of FeTP to DPPH. Ghlissi et al. reported that the antioxidant effect of biosynthesized nanoparticles is associated with the different types of surface adsorbed plant constituents which further contribute to accelerating the rate of wound closure and fast re-epithelialization (Ghlissi et al., 2016). IC_50_ of standard (ascorbic acid) was observed to be 0.74 μg/ml.

### Acute Dermal Toxicity Study

A new agent can cause a variety of undesired effects right from the dermal toxicity on topical application. FeTP contains a mixture of components with different polarities which may affect the cutaneous tissues or cross different membrane barriers to reach the circulation system leading to physiological damage.

The current study is the first to explore the acute dermal toxicity of FeTP. Daily visual observation for 14 days confirmed the absence of any visible sign of dermal toxicity and other adverse reactions such as erythema, pruritus, inflammation and edema. No mortality was observed with the limit test dose.

### Effect on Rate of Wound Contraction

**FIGURE 4:**
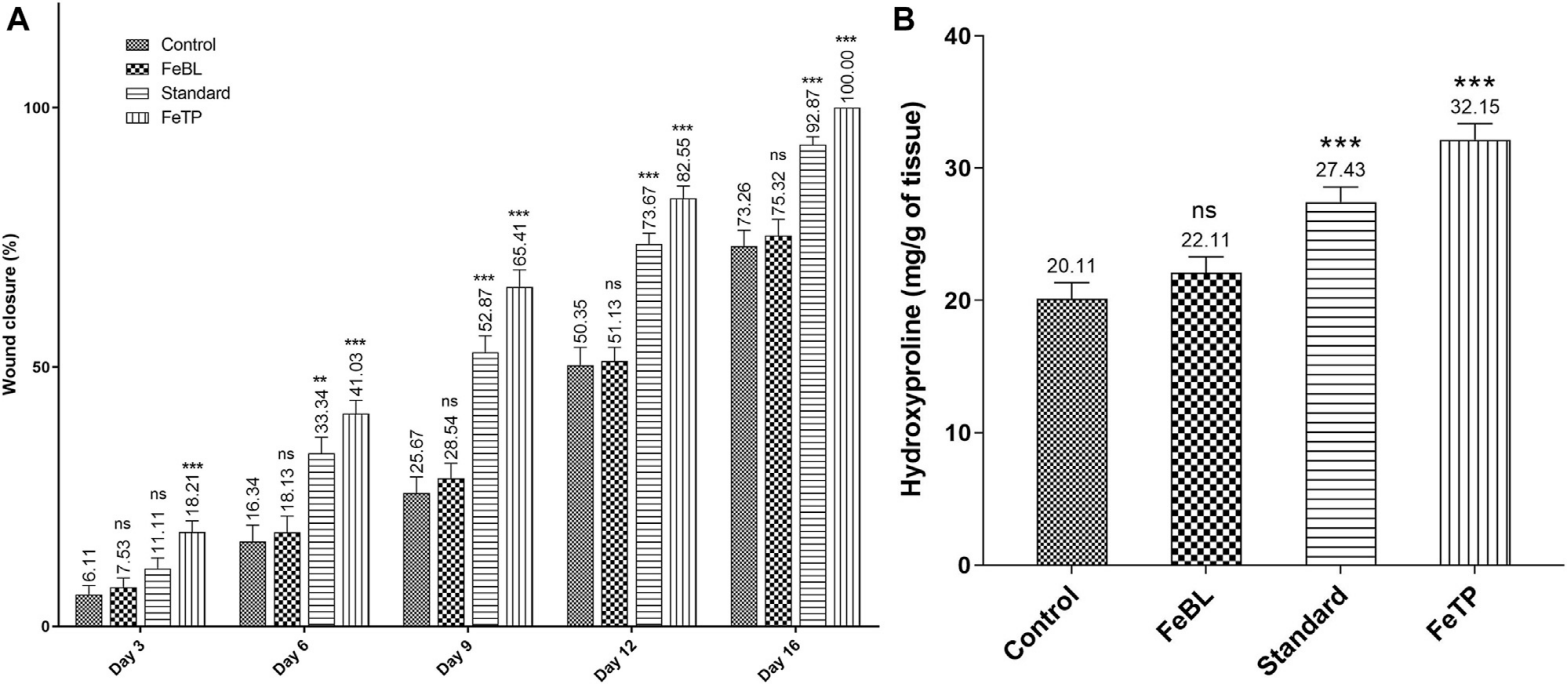
Effect of FeTP on excision wound expressed as **(A)** % wound closure and **(B)** hydroxyproline content. Values are represented as mean ± SEM (n = 6). Data were analyzed by one-way ANOVA followed by Dunnett’s test. Mean values are shown above bar and error bar. Significant difference **p < 0.01, ***p < 0.001, and ns: non-significant in comparison to control group.

Contraction of wound area was observed in FeTP, FeBL and standard groups on every third day as shown in (Figure 4A). Significant wound closure was noticed on third day post wounding only in FeTP applied group (*p* = 0.0008) compared to control group. Presence of granulation tissue on the excised area with minor inflammation was noticed on 6^th^ day in each group. The largest open wound area was observed in control group by accounting wound contraction rate of 16.34 ± 3.20%. On day 9 and 12, the wound contour was closed at a faster rate in FeTP group [day 9 (65.41 ± 3.33%, *p* < 0.0001); day 12 (82.55 ± 2.35%, *p* < 0.0001)] with more granulation tissue and reduction in visible inflammation as compared to control group while moderate wound closure effect was noticed in standard group and non-significant effect (*p* = 0.8521, *p* = 0.9937) was shown by FeBL group. Complete healing of injured tissue was observed in FeTP group (100%, *p* < 0.0001) followed by standard group (92.87 ± 1.52%, *p* < 0.0001) with a slightly open wound area on 16^th^ day when compared to control group. The slowest rate of wound contraction with a visible sign of inflammation and widest wound area was observed in control group (73.26 ± 3.11%) on 16^th^ post-wounding day.

It has been reported that oxidative stress and inflammation are key factors attributed to impediments in the wound healing process (Yadav et al., 2018b). Under the condition of oxidative stress, the excessive level of free radicals gets accumulated at wound site that promotes inflammation and pus formation which further leads to an increase in time of wound contraction (Seydi et al., 2019). It has been reported that FeNP exhibits favorable effects in the proliferative phase (collagen synthesis, angiogenesis, re-epithelialization) as well as remodeling phases (formation of collagen I, ECM remodeling, scar formation) of wound repairing process (Naskar and Kim, 2020). TP has been reported to possess a huge amount of phenolic compounds that promote wound contraction by contributing to collagenation and epithelialization of wound as it has antibacterial, anti-inflammatory and antioxidant properties (Yadav et al., 2017). Therefore, wound healing potential of FeTP may be credited with the synergistic effect of FeNPs as well as surface adsorbed phenolic compounds of TP.

### Hydroxyproline Content

Hydroxyproline is one of the essential components of the triple-helix strand of collagen fiber, therefore, hydroxyproline content is known as an index of collagen synthesis (Goldberg and Green, 1969). High concentration of hydroxyproline directs the cell proliferation as well as synthesis, localization and maturation of collagen which predominantly delineates the rate of wound healing (Harris and Fraser, 2004). Results of the current study revealed that FeTP group (*p* < 0.0001) and standard group (*p* = 0.0009) have a significantly high level of hydroxyproline when statistically compared with control group. FeBL group (*p* = 0.5120) hydroxyproline content was found to be non-significant as compared to control group (Figure 4B). Results revealed that topical treatment of wound with FeTP promotes healing by advancing collagen synthesis via hydroxyproline content.

### Granulation Tissue’s Enzymatic Antioxidant Status

**FIGURE 5:**
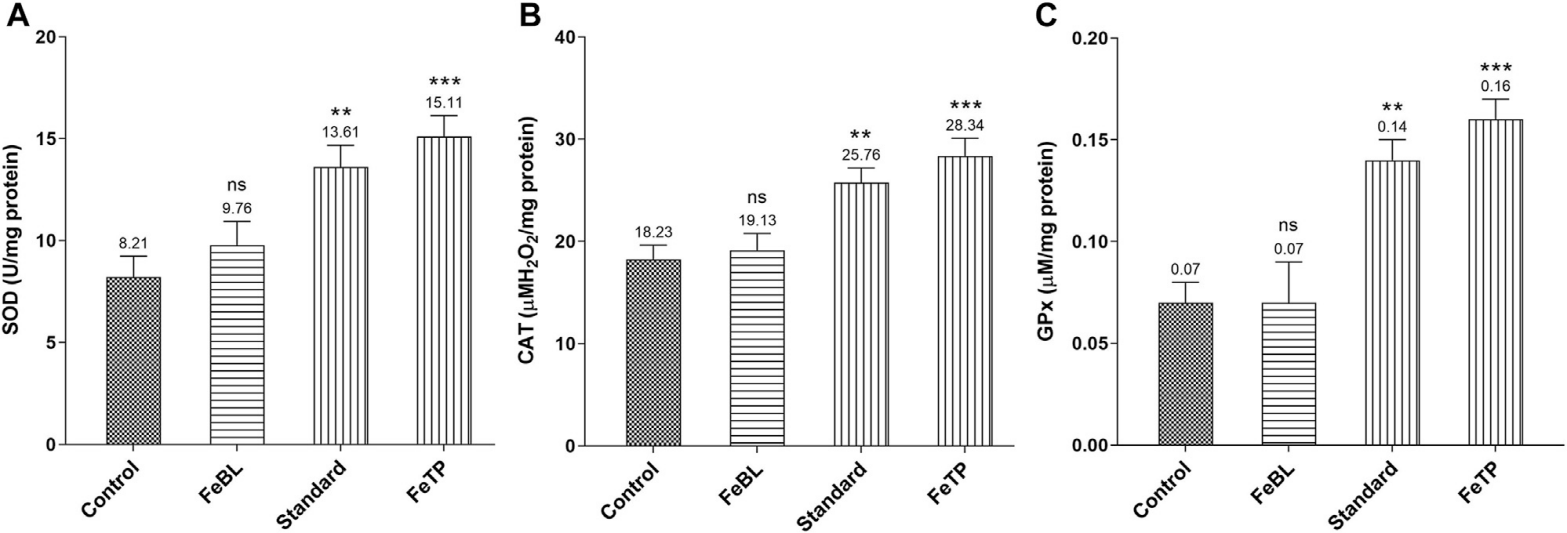
Effect of FeTP on antioxidant enzyme profile in granulation tissue by estimation of **(A)** SOD level, **(B)** CAT level, and **(C)** GPx level. Data represented as mean ± SEM, analyzed by one-way ANOVA followed by Dunnett’s test. Mean values are shown above bar and error bar. Significant difference **p < 0.01, ***p < 0.001, and ns: non-significant in comparison to control group.

The optimum amount of free radicals secreted by macrophage and neutrophils at the time of inflammatory phase of wound healing gets neutralized by the body’s defense system endogenously. But over generated free radicals affect redox hemostasis, fibroblast proliferation and vital functions of surrounding tissue reversibly or irreversibly result in thwarted wound healing (Yadav et al., 2018b). In our body, SOD and CAT are endogenous cellular antioxidants that transform superoxide into hydrogen peroxide (H_2_O_2_) and molecular oxygen. H_2_O_2_ is further neutralized by peroxidases (CAT and GPx) into water molecules (Kurahashi and Fujii, 2015). Therefore, inhibition of ROS overproduction could be a promising tool for the swift healing of wounds. Considering the fact, level of tissue antioxidant enzymes were measured and a significantly reduced level of SOD, CAT and GPx were observed in control group due to ROS accumulation in the wound region. Continuous application of FeTP strongly increased the level of SOD, CAT and GPx (*p* = 0.0006, *p* = 0.0005, *p* = 0.0003 respectively) (Figure 5A–C). Standard group also exhibited significantly (*p* = 0.0055, *p* = 0.0077, *p* = 0.0035, respectively) elevated activity of these antioxidant enzymes compared to control group.

### Inflammatory Markers

**FIGURE 6:**
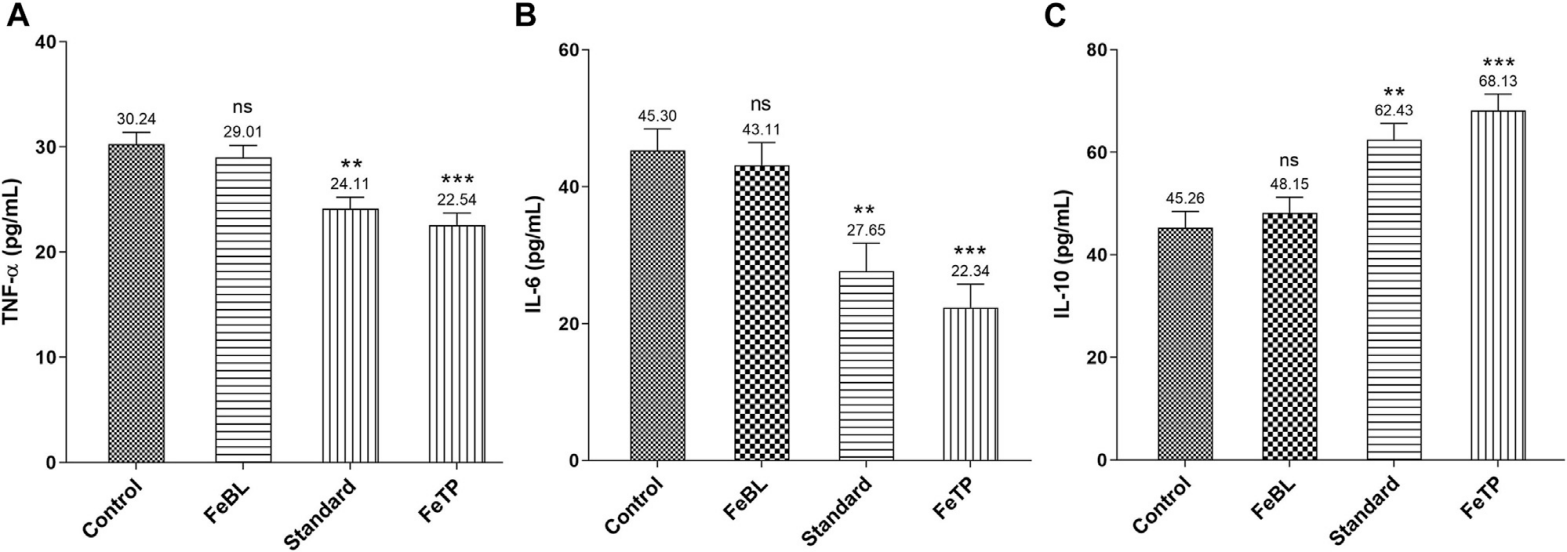
Effect of FeTP on inflammatory markers in granulation tissue by estimation of **(A)** TNF-α level, **(B)** IL-6 level, and **(C)** IL-10 level. Data represented as mean ± SEM, analyzed by one-way ANOVA followed by Dunnett’s test. Mean values are shown above bar and error bar. Significant difference **p < 0.01, ***p < 0.001, and ns, non-significant in comparison to control group.

In a normal wound healing process, proinflammatory cytokine such as TNF-α is released at the wound site in response to platelet activation and synthesizes the macrophage. This exhibits an essential role of TNF-α in inflammation management. With the progression of wound healing the level of TNF-α decreases which leads to the initiation of granulation tissue formation. TNF-α accelerates the proliferation of fibroblasts and granulation tissue production required for effective wound repairing process via activation of matrix metalloproteinase-2 (MMP-2) (Han et al., 2001; Thanusha et al., 2018). TNF-α protects from pathogens but it also causes a damaging effect to the host if expressed in huge quantity than usual, especially in diseased conditions related to inflammation. Past studies demonstrated that reduction in TNF-α level is concerned with an increase in collagen deposition which shows that this specific tissue cytokine is able to hinder the process of wound healing (Thanusha et al., 2018). Further, release of other important multifunctional cytokines, i. e. IL-6 and interleukin-1β (IL-1β), causes a delay in the epithelialization and granulation process by inhibiting the proliferation and migration of fibroblasts and keratinocytes during inflammatory and immune responses (Tanaka et al., 2014). Whereas IL-10, an anti-inflammatory cytokine, along with several growth factors such as Transforming Growth Factor (TGF-β) activated during inflammatory process is responsible for inhibition of synthesis of active macrophages from several proinflammatory cytokines (TNF-α, IL-1β and IL-6). IL-6 is also engaged in process of angiogenesis, fibroblast proliferation and extracellular matrix synthesis (Beserra et al., 2020; Xiao et al., 2020). Therefore, a successful wound healing mechanism may be attributed to the alleviation of aggravated levels of involved proinflammatory cytokines and increased anti-inflammatory cytokines expression. Results revealed a significant reduction in TNF- α and IL-6 level in FeTP group (*p* = 0.0003, *p* = 0.0005) as compared to control group (Figure 6A,B). While IL-10 level in FeTP group (*p* = 0.0002) was significantly increased compared to control group (Figure 6C). Our results are in well agreement with a previous study which demonstrated that inhibition of GSK activity in human monocytes significantly elevated the production of the anti-inflammatory cytokine, i. e. IL-10, while reduced the level of pro-inflammatory cytokines such as IL-1β, IL-6 and TNF-α (Martin et al., 2005). Our results exhibited that FeTP treatment is associated with favorable modulation of different tissue inflammatory cytokines (TNF-α and IL-6) concentration and acts as a potential wound healing agent as it owes an anti-inflammatory effect.

### Histopathological Examination

**FIGURE 7:**
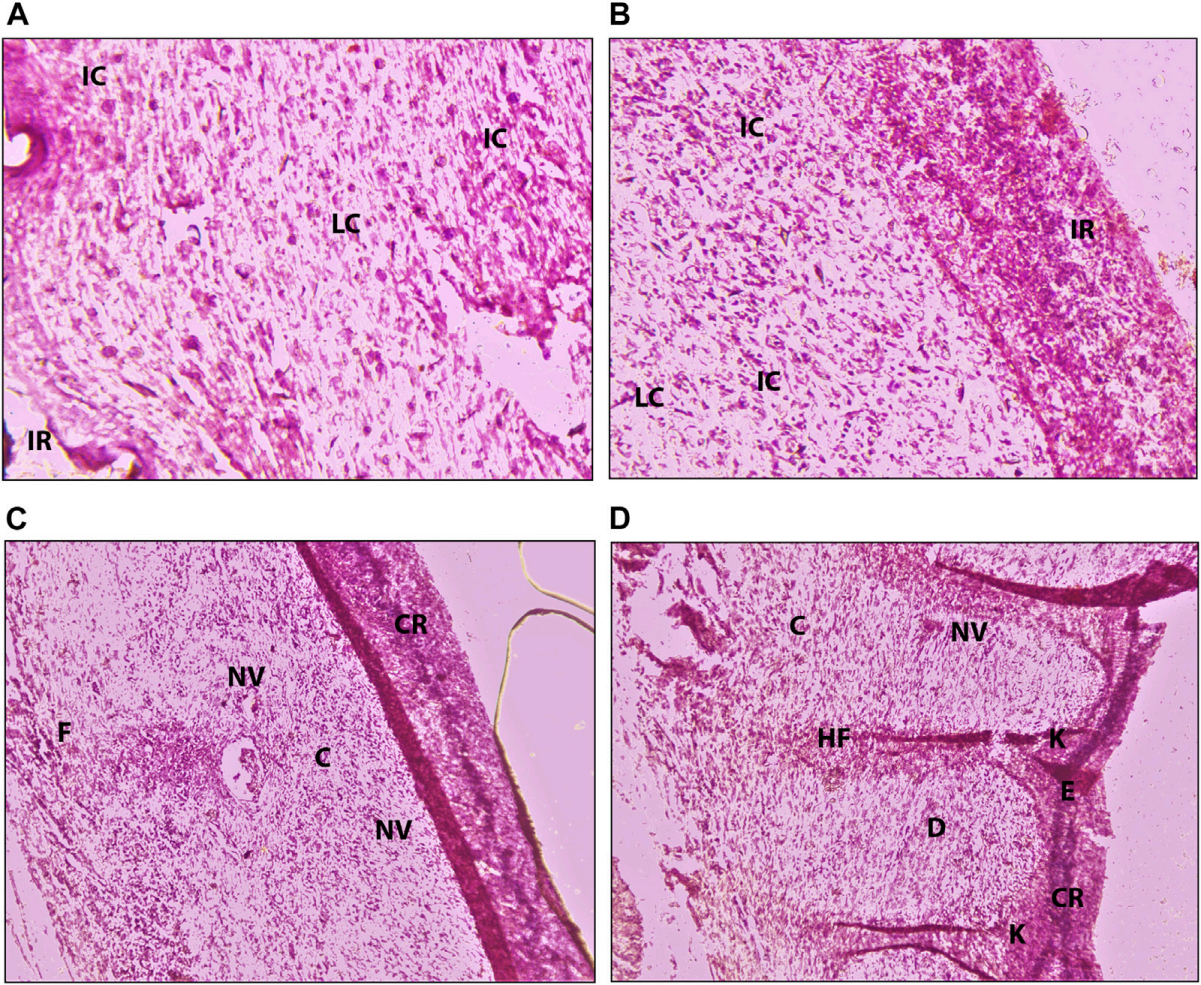
Photomicrograph (200X) of hematoxylin-eosin stained histological dermal section of wound area for **(a)** control group, **(b)** FeBL group, **(c)** standard group, and **(d)** FeTP group. C-collagen, CR-complete re-epithelialization, D-Dermis, DE-Disrupted epidermis, F-fibroblast, HF-hair follicle, IC-Inflammatory cells, IR- Incomplete re-epithelialization, K-keratinocytes, LC-Less amount of collagen, NV-neovascularization, PC-pus cells, SE-Separation of epidermis from dermis.

To confirm the complete wound healing effect of FeTP, histological studies of collected granulation tissues were assessed (Figures 7A–D; Table 2). As the healing process precedes, various changes were considered such as an increase in granulation tissue thickness, deposition of collagen fibers, the extent of re-epithelialization and reduction in inflammatory cells. The histopathological assessment showed parallel and oblique collagen fibrils, fibroblasts, a high number of pus cells and incomplete re-epithelialization in control group whereas rats treated with FeTP ointment exhibited horizontal and well-organized collagen fibers devoid of pus cells, complete re-epithelialization, hair follicles and keratinocytes that indicates faster and complete wound healing along with increased tensile strength. Collagen has the ability to maintain anatomical and physiological integrity of extracellular matrix and formation of new tissue. It is emphasized that collagen synthesis and its organization are some of the most imperative steps for wound healing (Ruszczak, 2003; Aszódi et al., 2006). Slide of standard group also showed improved tissue architecture with the sign of wound healing accounted with a good amount of collagen, few inflammatory cells, neovascularization, and reformation of the dehiscent epidermis. FeBL group showed the incomplete formation of dermis and epidermis as well as fewer amount of pus cells and fibroblasts.

**TABLE 2:** Histological data of the granulation tissue obtained from different groups.

Groups	Epidermal and dermal regeneration	Collagen formation	Angiogenesis	Inflammatory cells	Thickness of granulation tissue
Control	−	+	++	+++	+
FeBL	+	++	++	++	++
Standard	+++	+++	+++	−	+++
FeTP	+++	+++	+++	−	+++

Hematoxylin and eosin stained wound skin sections were scored as -: Absence, +: Slight, ++: Moderate, +++: Extensive.

### Tensile Strength

**FIGURE 8:**
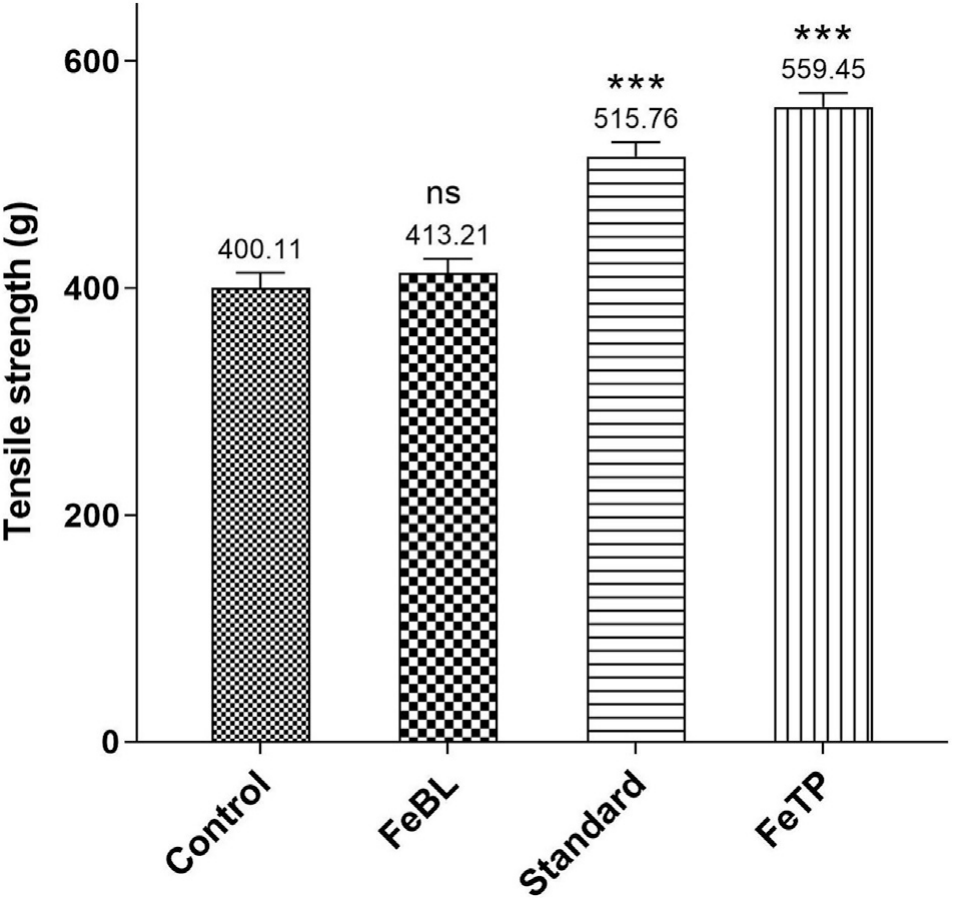
Effect of FeTP on tensile strength of newly healed tissue. Values are represented as mean ± SEM (n = 6). Data were analyzed by one-way ANOVA followed by Dunnett’s test. Mean values are shown above bar and error bar. Significant difference ***p < 0.001, and ns: non-significant in comparison to control group.

In the last phase of wound healing, remodeling of collagen occurs which results in the formation of collagen-I from collagen-III in the newly formed extracellular matrix followed by stable intra as well as inter-molecular cross-linking of fibers. This provides elasticity with high tensile strength to the newly healed tissue (Fikru et al., 2012; Naskar and Kim, 2020). Therefore, Tensile strength is considered as a marker of the breaking force of newly repaired tissue after injury. FeTP and standard group displayed a significant effect on breaking strength at the point of incision (Figure 8). On day 13, high tensile strength was exhibited by FeTP group (*p* < 0.0001) and standard group (*p* < 0.0001) as compared to control group. Rich formation and maturation of collagen at the site of wound attributed to high tensile strength and faster wound healing effect of FeTP.

## Conclusion

FeTPs were biosynthesized by the co-precipitation method. Characterization by using different analytical techniques confirmed the formation of 80–100 nm spherical shape FeTP. The existence of bioactive phytoconstituents of TP as a capping agent in FeNPs was detected with the help of FT-IR. Docking studies exhibited binding of the existed phytoconstituents against one of the imperative target proteins of the wound healing process, i. e. inhibition of GSK protein associated with Wnt/β-catenin signaling pathway. This substantiates the promising wound healing effect of biosynthesized FeTP *via* a significant antioxidant and anti-inflammatory potential. However, the precise mode of action responsible for the wound healing potential of FeTP may be evaluated at the cellular and molecular level in prospective future studies. An outcome of the present work unveils the new therapeutic approach for the treatment of wounds.

## Data Availability

The original contributions presented in the study are included in the article/Supplementary Material, further inquiries can be directed to the corresponding authors.
